# HIV and tuberculosis co-infection among migrants in Europe: A systematic review on the prevalence, incidence and mortality

**DOI:** 10.1371/journal.pone.0185526

**Published:** 2017-09-28

**Authors:** Ana Maria Tavares, Inês Fronteira, Isabel Couto, Diana Machado, Miguel Viveiros, Ana B. Abecasis, Sónia Dias

**Affiliations:** Global Health and Tropical Medicine, GHTM, Instituto de Higiene e Medicina Tropical, IHMT, Universidade Nova de Lisboa, UNL, Lisboa, Portugal; National Institute of Health, ITALY

## Abstract

**Background:**

International human migration has been rapidly growing. Migrants coming from low and middle income countries continue to be considerably vulnerable and at higher risk for infectious diseases, namely HIV (Human Immunodeficiency Virus) and tuberculosis (TB). In Europe, the number of patients with HIV-TB co-infection has been increasing and migration could be one of the potential driving forces.

**Objective:**

This systematic review aims to improve the understanding on the burden of HIV-TB co-infection among migrants in Europe and to assess whether these populations are particularly vulnerable to this co-infection compared to nationals.

**Design:**

MEDLINE^®^, Web of Science^®^ and Scopus^®^ databases were searched from March to April 2016 using combinations of keywords. Titles and abstracts were screened and studies meeting the inclusion criteria proceeded for full-text revision. These articles were then selected for data extraction on the prevalence, incidence and mortality.

**Results:**

The majority of HIV-TB prevalence data reported in the analysed studies, including extrapulmonary/disseminated TB forms, was higher among migrant vs. nationals, some of the studies even showing increasing trends over time. Additionally, while HIV-TB incidence rates have decreased among migrants and nationals, migrants are still at a higher risk for this co-infection. Migrants with HIV-TB co-infection were also more prone to unsuccessful treatment outcomes, death and drug resistant TB. However, contradicting results also showed lower mortality compared to nationals.

**Conclusions:**

Overall, a disproportionate vulnerability of migrants to acquire the HIV-TB co-infection was observed across studies. Such vulnerability has been associated to low socioeconomic status, poor living conditions and limited access to healthcare. Adequate social support, early detection, appropriate treatment, and adequate access to healthcare are key improvements to tackle HIV-TB co-infection among these populations.

## Introduction

The number of international human migratory movements worldwide has been growing over the past fifteen years, reaching 244 million in 2015 [1]. Since the 1960s, a steady increase in the number of international migrants coming to and living in Europe has been recorded [2]. Migration is, therefore, recognized as a key component of population change in Europe [3]. In 2015, 1,046,599 migrants arrived to Europe [2], and 76 million international migrants were residing in Europe, a huge increase compared with the year 2000 (56 million) [1].

Due to these increasing numbers, and regardless of the abiding movements and recent social awareness for the human crisis affecting Europe, migrants remain among the most vulnerable members of the European societies [1], and can be at risk for diseases, including infectious diseases, due to poor living conditions or other disparities [3]. In fact, in the European Union, migrant populations are at a greater risk of HIV and/or TB acquisition than the general population [4].

HIV and TB have been influencing each other’s natural history and pathogenesis over time, enhancing the magnitude of HIV-TB co-infection epidemic [5]. HIV infection is the strongest known risk factor for developing active TB, which is also the most common opportunistic disease among HIV-infected patients [6]. People living with HIV/AIDS (Acquired Immunodeficiency Syndrome) and infected with *Mycobacterium tuberculosis* (latent TB) are at twenty-times greater risk of developing active TB [6,7], and the intersection of both diseases contributes to a significant higher morbidity and mortality [6].

Globalization and migration from endemic zones have been considered a major drive in the global spread of HIV-TB co-infection [8]. In the European Region of the World Health Organization (WHO), the number of patients with HIV-TB co-infection increased between 2008 and 2014 [9], which some authors attributed partially to migration [10]. Social, economic and political factors in the origin and destination countries influence the risk of migrant populations to HIV acquisition—poverty, separation from sexual partners, different social and cultural norms, language barriers, substandard living and exploitative working conditions, including sexual violence—force many migrants to engage into risky behaviours, increasing the risk for acquiring the infection. Moreover, living and working conditions in the host country (access to health services and social protection), travelling journey to Europe (higher risk in crowded transport vehicles with poorly ventilated spaces plus unhealthy conditions in many migrant camps across the journey), TB incidence in their country of origin and previous contact with an infectious case, are determinant factors for TB infection among migrants [11].

Many countries have made considerable progress in addressing HIV-TB co-infection, but many global targets have not been reached yet [5]. Despite the importance of TB and HIV as public health problems in the European Region of the WHO [7,12], data available is limited on the risk factors for HIV-TB co-infection [12] and the case-reporting is often incomplete [13]. The available information on the HIV-TB co-infection burden among migrants living in Europe is still limited. This information is crucial to provide a comprehensive view to inform policies and improve adequate care and support to these populations. In this study, a systematic review of literature was conducted aiming to improve the understanding of the burden of HIV-TB co-infection among migrants in Europe and to compare the prevalence, incidence and mortality in this population with nationals in Europe. This systematic review is one of the first addressing specifically on the burden of this co-infection among migrants and the results obtained clearly demonstrate the importance for the national HIV-TB programs to address this reality systematically in order to control the predicted impact on these vulnerable populations and on the national control programs.

## Materials and methods

A combination of key words and/or Medical Subject Headings (MeSH) terms was used to find relevant studies. Our search was defined, using specific tools available in the searched databases, to retrieve publications between 2000 and 2016. Only articles with abstracts and written in English, Spanish, French or Portuguese were considered. Books or book chapters, comments, editorials, reviews, guidelines, reports, newspaper articles and case-studies were not included.

The electronic databases MEDLINE^®^, Web of Science^®^ and Scopus^®^ were systematically searched between March and April 2016 for original articles using search terms presented in S1 Table. MEDLINE^®^ was the first choice since it is one of the largest bibliographic databases focused on medical related fields [14,15]. Scopus^®^ was also searched as it includes also EMBASE^®^ database additionally to MEDLINE^®^ content, plus other journals indirectly related to the medical field [16]. Web of Science^®^ (via https://www.webofknowledge.com) was also included due to its coverage on medical or medically related journals missed by Pubmed and EMBASE^®^ [17].

The titles and abstracts of all documents retrieved were screened by one main reviewer (Ana Maria Tavares—AMT). A second reviewer (Inês Fronteira—IF) performed screening in a random sample of retrieved documents—the minimum sample size was calculated in OpenEpi platform (in www.openepi.com) using an anticipated frequency of 7.6%, for a 95% Confidence Interval (CI)—, in order to access sensibility and specificity of the inclusion criteria [18]. Disagreement between reviewers concerning this sample of documents was solved through reanalysis of the respective titles/abstracts and consensus.

Only the scientific papers meeting the following inclusion criteria were selected: 1) the studied population includes migrant subjects infected with HIV and TB, 2) it provides measures of prevalence, incidence and/or mortality; 3) the study and/or studied population was sampled in one or more European countries (of the 51 independent states [19]); 4) it is an observational study. The following exclusion criteria were defined: 1) articles in which the studied population does not mention human migrants (immigrants, emigrants and others); 2) articles with migrants not living in European countries; 3) articles about infectious diseases other than HIV and/or pulmonary tuberculosis; 4) articles about HIV or TB only, separately; 5) articles about co-infections other than HIV-TB co-infection; 6) articles without the outcomes of interest (prevalence, incidence and/or mortality).

After screening for titles and abstracts, the selected articles proceeded for fulltext review, in which, only articles meeting all inclusion criteria and not meeting any exclusion criteria were considered for data extraction. The data extraction process was performed by one researcher (AMT). Data on the prevalence, incidence and mortality associated with co-infection in migrants and nationals (when available) were extracted. Prevalence of extrapulmonary and/or disseminated TB and drug resistant TB among HIV-TB co-infection cases were also considered, as well as measures of risk and association related to HIV-TB co-infection in migrants.

For this systematic review no protocol was registered and no quality scoring system was applied.

## Results

A total of 746 articles were retrieved from databases (S1 Table) and, after removing duplicates (n = 251), 495 articles remained for title and abstract screening by one main reviewer (AMT). Of these, a sample of 214 articles was randomly selected for titles and abstracts screening by a second reviewer (IF).

**Fig 1:**
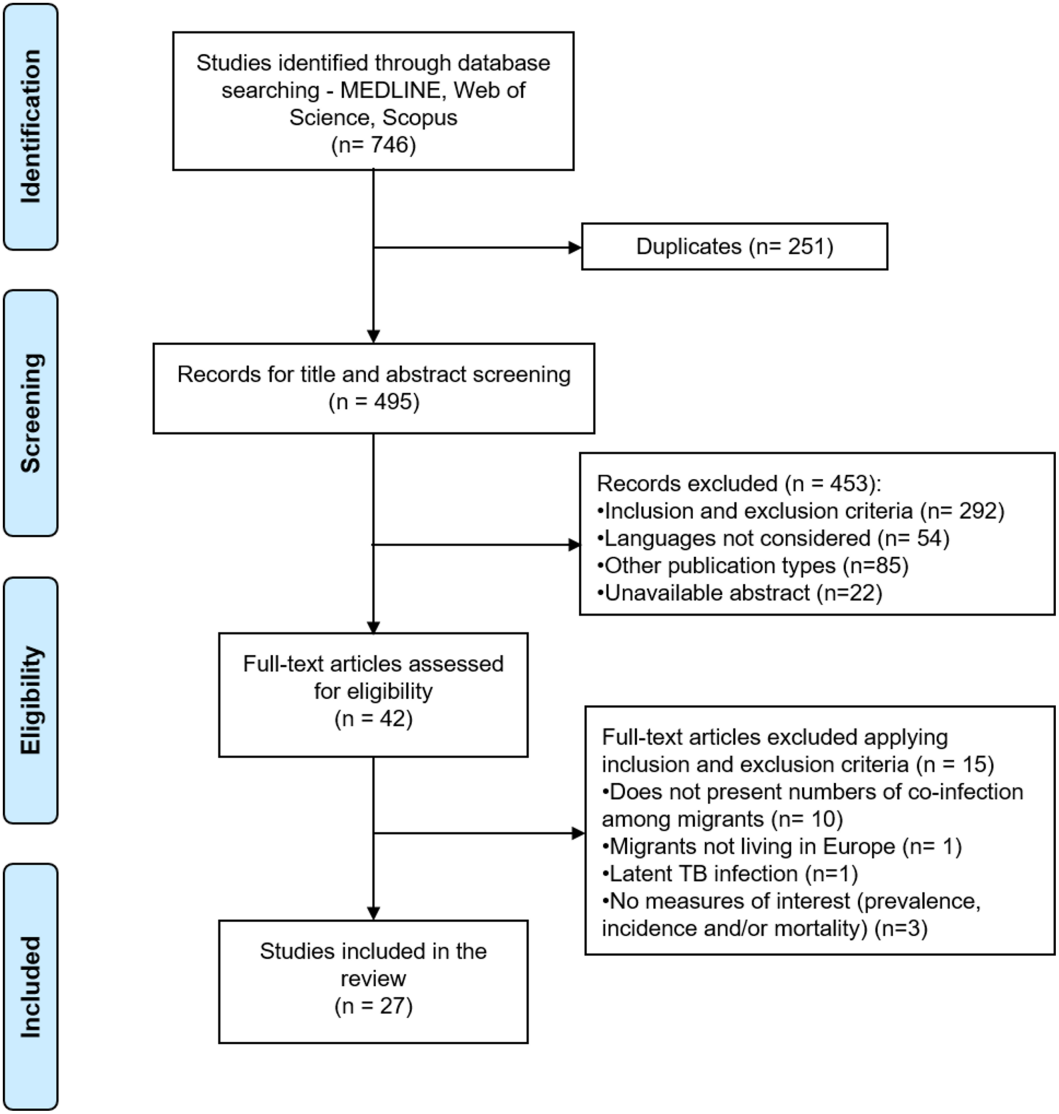
PRISMA flow diagram.

During screening, 453 articles were excluded: 292 articles were eliminated after applying the inclusion and exclusion criteria, 54 articles were written in other foreign languages not considered, 85 documents were publication types not considered for this review, and 22 documents lacked an abstract available for screening. After screening, 42 articles remained for fulltext revision and, after applying inclusion and exclusion criteria, only 27 articles were retrieved for data extraction. The full details of the articles selection process is summarised in Fig 1.

### Characteristics of the studies

The studies included were published between 2003 and 2016, while the sampling for those studies was conducted between 1984 and 2013. Eight European countries were represented: eleven studies conducted in Spain [20–30], five studies in Italy [31–35], three studies in France [36–38], two studies in Portugal [39,40], Germany [41,42], and United Kingdom (UK) [43,44], and one study in Switzerland [45] and The Netherlands [46] (Table 1).

**Table 1:** Main characteristics of the studies included in the review.

First author	Year	Year of data collection	Country	Sample			Type of study
				Type	Nr. Of subjects	Origin	
Abgrall et al. (a) [37]	2010	1997–2008	France	HIV patients	72580	France: 58 089 (80%); SSA: 9095 (12.5%); Others: 5396 (7.5%)	Prospective cohort study
Abgrall et al. (b) [36]	2010	1997–2008	France	HIV patients	72580	France: 58 089 (80%); SSA: 9095 (12.5%); Others: 5396 (7.5%)	Prospective cohort study
Baussano et al.[31]	2006	2001	Italy	New TB immigrant patients	640	EE: 43 (25%); Africa: 89 (52%); LA: 20 (12%); Asia: 13 (8%)	Population-based study
Brindicci et al.[32]	2016	2005–2013	Italy (BAT Province)	TB patients	129	Italy: 85; Immigrants: 44 immigrants [EE: 25 (22.7%); SSA: 10 (22.7%); NA: 6 (13.6%)]	-
Camoni et al.[33]	2012	1993–2010	Italy	HIV-TB patients	4075	Italy: 2685 (65.9%); Immigrants: 1390 (34.1%) (Africa: 55.3%; SA: 29.0%; EE: 7.9%; Asia: 5.7%; Others: 2.1%	-
Caro-Murillo et al.[20]	2009	2004–2006	Spain	HIV patients	2507	Spain: 1793 (71.5%); WE: 93 (3.7%); EE: 42 (1.7%); SSA: 145 (5.8%); NA: 34 (1,4%); LA/Caribbean: 400 (16.0%)	-
Diz et al.[21]	2007	1990–2002	Spain	Immigrant patients	1353	LA: 55%; Africa (37%).	-
Ennemoser et al.[42]	2015	1994–2013	Germany	HIV-TB/TB immigrant patients	47; 46	Africa: 53; Asia: 34; EE: 6	Retrospective study
Eszol et al.[22]	2009	2000–2006	Spain (Alicante)	immigrant HIV patients	69	LA: 38; SSA: 23; EE: 7; NA: 1	Retrospective study
Girardi et al.,[34]	2012	-	Italy	HIV-TB patients	246	Italy: 162; Foreign-born: 84	Multicenter prospective study
Karo et al.[41]	2014	2001–2011	Germany	HIV patients	11693	-	Cohort study
Kesselring et al.[46]	2010	1996–2008	Netherlands	Foreign-born HIV patients	6057	WE/North America: 3947 (65%); SSA: 989 (16%); Southeast Asia: 237 (4%); LA/Caribbean: 695 (11%); Others: 189 (3%)	Cohort study
Llenas-Garcia et al.[23]	2012	1992–2009	Spain (Madrid)	Immigrant HIV patients	371	LA: 197 (53.1%); SSA: 91 (24.5%); Caribbean: 32 (8.6%); EE/Central Asia: 20 (5.4%); Central-WE: 20 (5.4%); NA/Middle East: 9 (2.4%); North America: 1 (0.3%); South and Southeast Asia: 1 (0.3%)	Retrospective study
Martin et al.[24]	2011	1994–2005	Spain (Barcelona)	AIDS patients	3600	Spain: 3279; Immigrants: 321	Retrospective study of prevalence
Meyssonier et al.[38]	2012	1995–2008	France	new TB patients	14610	France: 7481; Foreign-born: 7129 [SSA: 2770 (39%); Maghreb: 2101 (30%); Asia: 1243 (17%); Europe: 695 (9.8%) (EE/Balkans: 308 (44%); Central Europe: 53 (8%); WE: 334 (48%))].	-
Ortega et al.[25]	2007	2001–2005	Spain (Madrid)	Foreign-born HIV patients	78	SSA: 41 (56.9%); SA: 19 (26.4%); Others: 18 (16.7%)	-
Ospina et al.[26]	2012	2000–2002 and 2003–2005	Spain (Barcelona)	Foreign-born TB patients	572 (2000–2002); 388 (2003–2005)	2000–2002 –LA: 202 (35.3%); India/Pakistan: 136 (23.8%); NA: 92 (16.1%); Others: 142 (24.8%). 2003–2005 –LA: 152 (39.2%); India/Pakistan: 112 (28.9%); NA: 42 (10.8%); SSA: 16 (4.1%); Others: 66 (17%)	Quasi-experimental study
Paulino et al.[39]	2016	2008–2012	Portugal	native-born TB patients; foreign-born TB patients	4131; 2009	Nationals: 4131; Foreign-born: 2009 [Africa: 1484 (73.9%); SA: 209 (10.4%); EE: 197 (9.8%); Asia: 104 (5.2%); Others: (0.7%)]	Retrospective study
Rajamanoharan et al.[44]	2004	2001–2002	United Kingdom	Persons with insecure immigration/ seeking asylum	-	-	-
Ramos et al.[27]	2004	1999–2002	Spain (Elche)	TB patients	105	Nationals: 83; Immigrants: 22 [Morocco: 5 (22.7%); SA: 9 (40.9%); EE: 4 (18,2%); SSA: 4 (18,2%)]	-
Rice et al.[43]	2013	2002–2010	England and Wales	HIV-TB patients	45322	Foreign-born: 3163 (96% - 3163/in 3310 patients co-infected)	Population-based register
Rifes and Villar[40]	2003	1996–2000	Portugal (Amadora)	TB patients	1013	Nationals: 765; Immigrants: 248 [Cape Verde: 107 (43,1%); Angola: 60 (24,2%); Guinea: 40 (16,1%); S.Tome and Principe: 21 (8,5%); Mozambique: 12 (4,8%); Timor: 1 (0,4%); Others: 7 (2,8%)	Retrospective study
Rodriguez-Valin et al.[28]	2015	2012	Spain	TB patients	5880	Nationals: 3992; Foreign-born: 1888	Retrospective study
Scotto et al.[35]	2006	2003	Italy	immigrant patients	2392	Africa: 145 (48.3%); Asia: 60 (20%); EE: 61 (20.3%); SA: 34 (11.3%)	Multicentric study
Staehelin et al.[45]	2003	1989–2001	Switzerland	HIV immigrant patients	11872	Northwestern Europe: 9420 (79%); SSA: 671 (6%); Others: 1781 (15%).	Prospective national cohort study
Supervía et al.[29]	2015	2006–2012	Spain (Barcelona)	new TB immigrant patients	94	Asia: 49; LA: 45	Retrospective descriptive study
Velasco et al.[30]	2008	1984–2000	Spain (Madrid)	HIV-TB patients	1284	Nationals: 1185; Immigrants: 99 [Africa: 62.6%; Central/SA: 16.2%; EE: 4%; WE: 14%; Asia: 3%].	-

BAT—Barletta-Andria-Trani; EE- Eastern Europe; LA—Latin America; NA—North Africa; SA—South America; Sub-Saharan Africa—SSA; WE—Western Europe

The main study design was retrospective—eight studies [22–24,28,29,39,40,42]—followed by six prospective/cohort studies [34,36,37,41,45,46] (one of them also multicentric [34]), two population-based studies [31,43], one quasi-experimental study [26] and one multicentric study [35]. The remaining studies did not mention the adopted study design [20,21,25,27,30,32,33,38,44] (Table 1). Four studies provided data from national registries [28,33,39,43].

The sample size ranged between studies from 69 [22] to 72580 subjects [36,37]. The included subjects varied between studies: some studies included patients diagnosed with TB [27,28,32,38–40], HIV/AIDS [20,24,36,37,41,46], or HIV-TB co-infection [30,33,34,43]. Other studies included immigrant patients [21,35], immigrant/foreign-born TB patients [26,29,39,42], immigrant/foreign-born HIV patients [22,23,25,45,46], and immigrant/foreign-born HIV-TB patients [42].

Considering the region of origin, Africa was predominant, with higher percentages of immigrants born in Africa in 10 retrieved studies [25,30,33,35–37,39,40,42,46], followed by Latin America [20–22,26,27], and Western/Eastern Europe [38,45] (Table 1).

### Prevalence of HIV-TB co-infection among migrants

Prevalence measures of HIV-TB co-infection were reported in 20 of the 27 studies selected in this review (Table 2).

**Table 2:** Prevalence of HIV-TB co-infection among national and migrant patients.

References	Year	Country	Sample	Prevalence of HIV-TB cases	
				Nationals—n (%)	Migrants—n (%)
**Abgrall et al. (a)** [37]	2010	France	HIV patients	1394 (2.4%)	1231 (8.5%)
**Baussano et al.**[31]	2006	Italy	New TB immigrant patients	NA	32 (5%)
**Brindicci et al.**[32]	2016	Italy (BAT Province)	TB patients	9.4% HIV-TB (p>0.05)	6.8% HIV-TB (p>0.05)
**Caro-Murillo et al.**[20]	2009	Spain	HIV patients	41 (2.3%)	4 (2.8%) from SSA; 12 (3%) from LA/Caribbean; 3 (3.2%) from WE
**Diz et al.**[21]	2007	Spain	Immigrant patients	37%	6% (p<0.001) (14% from Africa, 2% from LA)
**Ennemoser et al.**[42]	2015	Germany	HIV-TB and TB immigrant patients	NA	47 (51%): (higher proportion of patients from Africa [36 (76.6%]
**Eszol et al.**[22]	2009	Spain (Alicante)	Immigrant HIV patients	NA	8 (11.6%): 4 (17.4%) from SSA, 1 (2.6%) from LA, 3 (42.8%) from EE.
**Kesselring et al.**[46]	2010	Netherlands	Foreign-born HIV patients	NA	58 (1%): (higher proportion in patients from SSA vs. WE/North America—3.0% vs. 0.4%)
**Llenas-Garcia et al.**[23]	2012	Spain (Madrid)	Immigrant HIV patients	NA	36 (9.7%): 13.2% from SSA, 33.3% from North Africa/Middle East, 6.1% from LA, 6.3% from Caribbean, 20% from Central/WE, 10% from EE/Central Asia
**Martin et al.**[24]	2011	Spain (Barcelona)	AIDS patients	30.8% (p = 0.02)	37.1% (p = 0.02): 50% from North Africa/Middle East, 50% from SSA, 31.7% from LA/Caribbean, 36.4% from South Asia/East Asia/Pacific, 29.9% from WE/North America, 45.5% from Europe/Central Asia
**Meyssonier et al.**[38]	2012	France	New TB patients	6.5%	11%
**Ortega et al.**[25]	2007	Spain (Madrid)	Foreign-born HIV patients	NA	16 (20.5%)
**Ospina et al.**[26]	2012	Spain (Barcelona)	Foreign-born TB patients	NA	49 (8.6%) in 2000–2002, 36 (9.3%) in 2003–2005
**Paulino et al.**[39]	2016	Portugal	Native and foreign-born TB patients	671 (16%)	452 (22%)
**Rajamanoharan et al.**[44]	2004	United Kingdom	Persons with insecure immigration/ seeking asylum	15% (p<0.001)	85% (p<0.001)
**Ramos et al.**[27]	2004	Spain (Elche)	TB patients	12 (14.5%) (p = 0.4)	2 (9.1%) (p = 0.4)
**Rifes and Villar**[40]	2003	Portugal (Amadora)	TB patients	182 (18%)	66 (26.6%)
**Scotto et al.**[35]	2006	Italy	Immigrant patients	NA	31 (10.3%): 18 from Africa, 8 from LA, 3 from EE, 2 from Asia,
**Staehelin et al.**[45]	2003	Switzerland	HIV immigrant patients	NA	7 (1%) (from SSA)
**Supervía et al.**[29]	2015	Spain (Barcelona)	New TB immigrant patients	NA	5 (11.1%) (from LA)

BAT—Barletta-Andria-Trani; EE- Eastern Europe; NA- not applicable; SSA- Sub-Saharan Africa; LA—Latin America; WE—Western Europe

Among these, 10 studies reported prevalence numbers of HIV-TB co-infection in immigrants and nationals [20,21,24,27,32,37–40,44]. Prevalence of co-infection was higher among immigrants than among nationals in 7 studies conducted in France, Portugal, Spain and UK [20,24,37–40,44]—range of 2.8%-85% among migrants vs. 2.3%-30.8% among nationals (Table 2)—, of which, one study was based on national registries from Portugal (2008–2012) [39]. Contradictory results were observed in 3 studies [21,27,32]—ranging between 6%-6.8% among migrants vs. 2.3%-37% among nationals (Table 2). Studies including only migrant patients, most conducted in Spain, showed prevalences of HIV-TB co-infection ranging from 1% to 76.6% [22,23,25,26,29,31,35,42,45,46] (Table 2).

Increases in prevalence of HIV-TB co-infection among migrants during data collection periods were reported in 3 studies. In the city of Barcelona, a significant increase was observed in the prevalence of HIV-TB co-infection in migrants, from 6.5% in 1994 to 37.1% in 2004, contrarily to nationals, in which a significant decrease has been observed [24]. Another study in Barcelona also reported a small increase in the prevalence of HIV-TB co-infection among migrants from 8.6% in 2000–2002 to 9.3% in 2003–2005 [26]. This increasing trend was also observed in the UK in the number of HIV-TB cases either among persons with insecure immigration or seeking asylum, from 45 in 2001 to 78 in 2002 [44].

Ten studies reported prevalence of HIV-TB co-infection per migrants’ region of origin [20–24,29,35,42,45,46], namely African [20–24,35,42,45,46], Latin American [20–24,29,35], European [20,22,23,35], and Asian regions [24,35]. The highest HIV-TB percentages were observed in migrants from African regions (range 1%-76.6%) [42,45], particularly migrants from SSA (range 1%-50% [24,45]), followed by migrants from Europe [3.2% (Western Europe) - 42.8% (Eastern Europe) [20,22]], from Asia (36.4% from South Asia/East Asia/Pacific [24]), and from Latin America (range 2% to 31.7% [21,24]) (Table 2).

Concerning the prevalence of various TB forms among HIV infected patients, seven studies reported extrapulmonary and/or disseminated TB cases [20–23,25,30,35], of which, three, all conducted in Spain, compared prevalence between migrants and nationals. Higher percentage of extrapulmonary TB was reported among HIV-infected migrants (75.8% vs. 68.4% in nationals) from 1984 to 2000, however non-significantly [30]. Contradictory results were observed between 1990 and 2002, with a higher rate of disseminated TB among HIV-infected nationals (33%) [21]. However, between 2004 and 2006 a significantly higher percentage of extrapulmonary TB was observed among HIV-infected migrants from Eastern Europe/Russia, Sub-Saharan Africa, Western Europe, North Africa, and Latin America/Caribbean (9.5%, 5.5%, 4.3%, 2.9%, and 2%, respectively vs. 2.5% in nationals) [20].

In Italy, a study including only migrants reported 13% of lymph node TB, 9.7% of multiple localization TB; 3.2% of osteoarticular TB, 3.2% of central nervous system TB, and 3.2% of intestinal TB in 2003 among HIV-TB infected migrants [35]. In Spain, studies performed in Alicante and Madrid, reported similar figures of disseminated TB—5.8% and 7.7%, respectively—, from 2000 to 2006 [22,25]. Another study in Madrid reported 37.1% cases of disseminated TB, 14.3% cases of ganglionar TB, 5.7% cases of tuberculous meningitis and 2.9% cases of pleural TB from 1992 to 2009 [23].

Among the included studies, 4 analysed proportion of migrants among co-infected cases [30,33,41,43]: two showing higher percentages of immigrants among co-infected patients [41,43], and two showing increasing trends in the proportion of migrants among co-infected patients during data collection [30,33].

Only a study in France reported prevalence of drug resistant TB among HIV-TB co-infected migrants and nationals, with a significantly higher percentage of resistance to streptomycin, isoniazid, rifampicin among foreign born patients compared to nationals (12.5%, 10.4% and 3.6%, vs. 8.0%, 6.7% and 1.2%, respectively) [38].

### Incidence of HIV-TB co-infection among migrants

Incidence rates of HIV-TB co-infection among migrants were reported in 6 studies [24,33,36,37,41,46]. Two studies conducted in France by the same authors on the same patients’ cohort reported a higher incidence rate among migrants—1.03/100 person-years; 95% CI: 0.95–1.11 vs. 0.28/100 person-years; 95% CI: 0.26–0.30 in nationals [36,37], despite of a significantly higher proportion of incident cases among nationals—564 (55.6%) vs. 330 (48.6%) in migrants—observed between 1997 and 2008 in one of the studies [37]. In the same two studies, the adjusted incidence rates showed an increase in the incidence of HIV-TB co-infection either among migrants (0.77/100 person-years in 1997; 1.60/100 in 2000; 1.24/100 person-years in 2002; 1.94/100 in 2008) and among nationals (0.46/100 person-years in 1997 person-years; 0.57/100 in 2000; 0.64/100 in 2002; 0.86/100 in 2008) during the study period [36,37]. Similarly, in Italy a higher incidence rate was observed among migrants—2.97/100 000 person years vs. 0.11/100 000 person years among nationals—, with a decrease over time among migrants (5.16/100 000 person-years in 1993 to 1.20/100 000 person-years in 2010) and nationals (0.17 /100 000 person-years in 1993 to 0.05/100 000 person-years in 2010) [33]. In England and Wales, HIV-TB incidence was higher among foreign-born patients in 2002 (42.5/1000 person-years vs. 8.6/1000 person-years among nationals) and 2010 (10.9/1000 person-years vs. 83.3/1000 person-years among nationals), also showing a decline in the HIV-TB incidence between 2002 and 2010 either for foreign-born (decline in 74.3%) or national patients (decline in 61.2%) [43]. A study conducted in Barcelona also showed higher incidence rates among male immigrants aged 29–49 years (15.8 vs. 12.7/100000 national inhabitants aged 20–29 years; 41.8 vs. 37.5/100000 national inhabitants aged 30–39 years; 33.4 vs. 14.7/100000 national inhabitants aged 40–49 years) and female immigrants aged 40–50 years old (7.9 vs. 1.3/100000 national inhabitants aged 40–49 years; 4.7 vs. 0.4/100000 national inhabitants aged 50–59 years), with an average rate decrease of 20% per year between 1994 and 2005 among both nationals and immigrants [24].

Three studies compared the incidence rates within migrants’ region of origin. A study conducted in Germany reported a significantly higher incidence density rate of HIV-TB co-infection in patients from Sub-Saharan Africa (1.20/100 vs. 0.21/100 person years in nationals) and other countries (0.52/100 vs. 0.21/100 person years in nationals) between 2001 and 2011 [41]. Similarly, in a study conducted in The Netherlands, the cumulative TB incidence after 7 years of combined antiretroviral therapy (cART) treatment was higher among HIV-positive patients from Sub-Saharan Africa compared with HIV-TB patients from Western Europe/North America (4.5% vs. 0.5%) [46]. A study conducted on the region of Piedmont, Italy, with new cases of TB among immigrant patients, showed annual incidence rate ratios of HIV-TB co-infection among patients from low prevalence countries of 179.3/100 000; 95% CI: 88.7–269.9 population among patients < 50 years, and 681.6/100 000; 95% CI: 212.7–1150.5 population among patients ≥50 years. Among patients from higher prevalence countries the annual incidence rate ratios were of 1139.5/100 000; 95% CI 403.1–1857.9 population among patients <50 years, and no incident cases among patients ≥50 years [31].

### Mortality and survival among HIV-TB infected migrants

Mortality and survival measures were reported in four studies [28,30,34,41], all with data on migrants and nationals. A study conducted in Germany from 2001 to 2011 observed a significantly lower survival in co-infected patients from Sub-Saharan Africa, compared to co-infected nationals (93% vs. 99% among nationals) [41]. However, contrasting results were shown previously in a study conducted in Spain from 1984 to 2000, with a significantly better survival of co-infected immigrants (median 8.7 vs. 5.4 years among nationals) and also a significantly lower mortality rate (0.42 vs. 0.45 among nationals) [30]. Another study conducted in Spain in 2012 also showed lower percentage of deaths among immigrant HIV-TB patients (6.99% vs. 8.79% among nationals) [28]. Similarly, a study from Italy reported a lower percentage of deaths among co-infected foreign-born patients (8.3% vs. 17.9% among nationals), however without statistical significance [34].

### Indicators and trends of risk and association

Eleven studies reported measures of risk and/or association [22,24,28,31,34,36–39,41,46]. Two studies performed in France between 1997 and 2008 using the same patients’ cohort observed twice more risk of TB among HIV-infected migrants—adjusted risk ratio (aRR) = 2.01; 95% CI: 1.79–2.26 [36,37]. An increased risk of HIV-TB from 2000/2001 to 2008 among nationals and migrants was also observed in one of the studies—aRR = 1.85; 95% CI: 1.27–2.70 [37]. Also, a non-significant 21% risk increase among nationals (aRR = 1.21, 95% CI: 0.86–1.70) and a significant 49% risk increase among migrants (aRR = 1.49, 95% CI 1.04–2.14) were observed from 2002–2003 to 2008 in the other study [36].

Three studies evaluated the risk of HIV-TB acquisition considering the regions of origin [31,41,46]. A study conducted in The Netherlands between 1996 and 2008 observed a 5-fold higher risk of HIV-TB among immigrants born in Sub-Saharan Africa compared to immigrants from Western Europe or North America (Hazard ratio (HR) = 5.08, 95% CI: 2.22–11.60) [46]. Similarly, a study conducted in Germany between 2001 and 2011 showed that being born in Sub-Saharan Africa significantly rendered a higher risk for HIV-TB [HR = 4.05; 95% CI: 1.87–8.78 among patients who never started combination antiretroviral therapy (cART) and HR = 5.15; 95% CI 2.76–9.60 among patients on cART], as well as being born in other countries than Germany (HR = 2.22; 95% CI 1.18–4.20 among patients on cART) [41]. A study in the Italian region of Piedmont referred that an HIV-positive status appeared to promote TB among immigrants from low and high prevalence countries, with a higher risk among those originating from low prevalence countries—incidence rate ratio of 51.9; 95% CI: 30.2–89.4 vs. 11.4; 95% CI 5.8–22.5 among those originating from high prevalence countries [31].

Four studies reported associations between migration and HIV-TB co-infection. A study conducted in Barcelona from 1994 to 2005 observed an association between being born in Sub-Saharan Africa and having TB and AIDS defining illness—adjusted odds ratio (aOR) = 2.2; 95% CI: 1.2–4.6 [24]. However, another study from Spain, performed between 2000 and 2006, showed a strong significant association of HIV-TB co-infection with being born in Eastern Europe—OR = 8.55; IC 95%: 1.5–49.4—and a negative association with being born in Latin America—OR = 0.09; 95% CI: 0.01–0.89 [22]. In a study in Portugal conducted between 2008 and 2012, the odds of being a foreign-born TB case among the HIV-positive population was approximately double compared to nationals—OR = 2.137; IC 95%: 1.65–2.77 [39]. Moreover, the abovementioned study conducted in France from 1997 to 2008 observed a higher risk for HIV-TB co-infection among migrants from Sub-Saharan Africa—adjusted risk ratio (aRR): 2.16 (95% CI: 1.88–2.48)—and other regions—aRR: 1.83 (95% CI 1.57–2.14)—, compared to nationals [37].

A study conducted in Italy referred an association between being a migrant with HIV-TB and unsuccessful treatment outcomes (i.e. lost to follow-up, failure, being transferred out, default)—OR = 3.38, 95% CI 1.38–8.29) [34]. Similarly, in a study in Spain a higher association with potentially unsuccessful outcomes and death was observed among co-infected foreign-born patients than in nationals (OR = 1.7; 95% CI: 1.15–2.60 vs. OR = 1.6; 95% CI: 1.09–2.29 and OR = 3.2; 95% CI: 1.53–6.76 vs. OR = 2.7; 95% CI: 1.63–4.54, respectively) [28]. In a study conducted in France, an association between being a migrant with HIV-TB and having TB resistance to streptomycin (OR = 1.6; 95% CI: 1.3–2.0), isoniazid (OR = 1.6; 95% CI: 1.3–2.1) and rifampicin (OR: 2.9; 95% CI: 1.9–4.6) was also observed, whereas co-infection in French-born patients was only associated with rifampicin resistance (OR: 4.7; 95% CI: 2.1–10.5) [38].

## Discussion

In this systematic review we investigated the burden of HIV-TB co-infection among migrants comparatively to national populations.

The results have shown that migrant populations are disproportionately affected by HIV-TB co-infection when compared to nationals. The majority of the studies reporting prevalence of HIV-TB co-infection showed significantly higher values among migrants compared to nationals, and some studies also showed a higher prevalence of extrapulmonary/disseminated TB among HIV-infected migrants. Moreover, in all the studies in which prevalence fluctuations of HIV-TB co-infection were shown, most of them conducted in Spain, a more pronounced increasing trend was observed among migrants, whereas a decreasing pattern was observed in some national populations. These results are in line with a 2017 ECDC report, describing an increase in the absolute number of patients with HIV-TB co-infection in the European Region of the WHO from 11652 cases in 2011 to 16380 in 2015 [47]. As previously described in a systematic review conducted in 2011, the increasing trends of HIV-TB co-infection might be related to migration, especially in countries such as Spain and Italy [10], which were also the countries of the majority of our studies. Nevertheless, considering that the comparison of HIV-TB prevalences between nationals and migrants was only performed in 10 of the 20 articles reporting prevalence of HIV-TB co-infection, such findings must be interpreted with caution, as we cannot disregard that different findings could be observed if more studies compared prevalence between the two populations.

When considering the country of origin, the highest prevalences were observed in migrants originating from African regions. It has been documented that HIV epidemic among the communities of sub-Saharan African migrants in Europe partially resembles the magnitude of the HIV epidemics in their home countries [48]. Also, the described reasons underlying the burden of TB among migrants are the interaction of migration from high TB burden countries and the reactivation in host countries [49]. Therefore, these prevalences might be related with origin from high HIV and TB prevalence countries in Africa, especially those from Sub-Saharan region. However, more data regarding regions of origin could provide a clearer view.

The incidence rates of HIV-TB were also shown to be significantly higher among migrant populations, as well as the risk for co-infection, affecting especially those from high prevalence regions, such as Sub-Saharan Africa. In fact, Africa is still the most common origin of migration to Europe and since the late 1980s there has been a hastening of emigration from this region to Europe [50]. Moreover, the prevalence of HIV-TB co-infection is the highest in the African region [51]. Therefore, it is not unexpected that migrants from Sub-Saharan Africa were observed to be at higher risk of co-infection in the analysed studies, since HIV co-infection has been found to be more likely in TB cases originating from Africa [47].

In this review, many studies reported a decrease in the incidence of HIV-TB co-infection over the data collection period among migrants and nationals. These promising findings may be interpreted as a success of control and prevention measures in Europe. However, a report from WHO refers that the incidence of HIV-TB co-infection has been slowly increasing since 1990 in the WHO European Region, being 2.2/100000 population in 2014 [51]. Therefore, no firm conclusions can yet be drawn based on these findings.

Being a migrant infected with HIV-TB was also associated with unsuccessful outcomes (treatment failure, being transferred out, and others), death and drug resistant TB, the later also observed in a previous review by Hargreaves et al. (2016) [52]. These findings are possibly related with factors influencing patients’ adherence to treatment, such as financial and social support, medication burden, side effects, stigma, beliefs and poor communication with health professionals [53]. Some of the studies [28,30,34] also referred that migrants co-infected with HIV-TB seemed to have lower mortality than nationals with the same co-infection. These results are quite contradictory in the light of the disproportionate vulnerability of migrants to HIV-TB observed in the previous results. Similar findings have been documented in a review by Domnich et al. (2012) and associated with the not yet fully understood and paradoxical “healthy migrant” effect—migrant populations may present a better health compared to nationals—, caused by a previous self-selection process prior to migration, in which only healthier and younger subjects are fit for emigration [54]. This effect would cause better chances of survival in case of infection, what could explain the lower mortality rates observed among migrants. However, according to Domnich et al. (2012), the “healthy migrant” effect is a temporary state that diminishes as time passes after immigration, possibly due to the disparities in the access to healthcare, and in the socioeconomic status [54]. These are factors that also might negatively impact the unsuccessful outcomes and TB resistance observed in our study. Although better survival was observed among migrants in this systematic review, Europe is one of the world’s regions with higher mortality rates caused by HIV-TB co-infection [55] and, therefore, it is important to understand the role of migration in HIV-TB associated mortality in European countries.

Methodological heterogeneity was observed in analysed studies, especially regarding study design, sample size, sampling procedure and epidemiological outcomes. Such differences rendered a challenging interpretation and comparison between studies.

Limitations of this systematic review must be acknowledged. Given the vast existing number of articles on the HIV, TB and/or migrants subjects, a narrow search strategy was used, very focused on the objectives of this review. MeSH terms were criteriously selected to be used in the MEDLINE^®^ database search, as well as restrictions for titles and abstracts search at Scopus^®^ database. Broader search terms could have also been used in the search expressions, such as “vulnerable populations” and “Europe”. We acknowledge that such methodological choices may imply a loss of comprehensiveness in our search. Also, the outcomes observed in the selected studies comprised different data collection periods, some of them taking place before the dawn of the combined antiretroviral therapy in 1996 [56]. In such studies, no distinction was made between data from pre and post-HAART period. No differences were observed when comparing the data from studies conducted before and after the introduction of HAART. Even so, we cannot exclude potential bias in the outcomes assessed in this review. Moreover, many retrieved studies were conducted on a specific region or city and, therefore, lack epidemiological representativeness of the problem in a certain country. In such cases, only a descriptive synthesis of evidence was possible and the findings must be interpreted only in the context of the represented region.

In this work we have been able to highlight the disproportionate vulnerability of migrants to acquire HIV-TB compared to nationals, a clear trend in the majority of the studies included. Higher prevalence, incidence, unsuccessful outcomes and drug resistance figures were observed among migrants living in European countries. The low socioeconomic status, the poor and overcrowded living and working conditions, malnutrition, substance use induced by marginalization, social exclusion [57], and barriers in the access to health care [58], are well described factors that may contribute for this disproportion between migrants and nationals. In order to tackle such inequities, European health systems must keep their efforts on the early detection and appropriate treatment of these infections among these populations, as well as to guarantee an adequate access to healthcare and efficient social support. Moreover, policies of inclusion and integration of these populations in the host society are of utmost importance in the preventive care of these diseases. Further research should continue on data collection from national registries on the HIV-TB co-infection among migrants, providing information on the epidemiological situation of each European country, and also in interventions to improve the main barriers to health care perceived by migrant patients infected with HIV-TB. Information on length of stay among migrants was also poorly explored in the included studies of this Systematic Review. Therefore, future research should also take into account this variable in order to allow better understanding of how the burden of HIV-TB co-infection varies with time of residency in the host country. This work highlights the importance for the national HIV-TB programs to thoroughly address this problem in order to mitigate the impact on these vulnerable populations and on the national control programs.

## Supporting information

S1 Table Database searches. (DOCX)

S2 Table PRISMA checklist. (DOC)
