# Supplementary material for: HIV and tuberculosis co-infection among migrants in Europe: A systematic review on the prevalence, incidence and mortality
Source: PLoS One. 2017 Sep 28;12(9):e0185526. doi: 10.1371/journal.pone.0185526 (PMC5619775; doi:10.1371/journal.pone.0185526)
Supplement: S1 Table — (DOCX) [file pone.0185526.s001.docx]

**S1 Table. Database searches.**

| **Database** | **Detailed Search Expression** | **Results** |
| --- | --- | --- |
| **MEDLINE (via PubMed)** | #1"Transients and Migrants/statistics and numerical data"[Mesh] OR "Emigrants and Immigrants/statistics and numerical data"[Mesh] OR "Human Migration/statistics and numerical data"[Mesh] OR "Human Migration/trends"[Mesh] OR immigration OR emigration OR non-resident OR non-native OR cross-border OR “labour migration” OR “international migration”; #2 Albania OR Andorra OR Armenia OR Austria OR Azerbaijan OR Belarus OR Belgium OR "Bosnia Herzegovina" OR Bulgaria OR Croatia OR Cyprus OR "Czech Republic" OR Denmark OR Estonia OR Finland OR France OR Georgia OR Germany OR Greece OR Hungary OR Iceland OR Ireland OR Italy OR Kazakhstan OR Kosovo OR Latvia OR Liechtenstein OR Lithuania OR Luxembourg OR Macedonia OR Malta OR Moldova OR Monaco OR Montenegro OR Netherlands OR Norway OR Poland OR Portugal OR Romania OR Russia OR "San Marino" OR Serbia OR Slovakia OR Slovenia OR Spain OR Sweden OR Switzerland OR Turkey OR Ukraine OR "United Kingdom" OR Vatican; #3 HIV OR "HIV Serosorting/statistics and numerical data"[Mesh] OR "HIV Infections/diagnosis"[Mesh] OR "HIV Infections/epidemiology"[Mesh] OR "HIV Infections/mortality"[Mesh] OR "HIV Infections/prevention and control"[Mesh] OR "HIV Infections/statistics and numerical data"[Mesh] OR "HIV Infections/transmission"[Mesh] OR "HIV Seroprevalence/trends"[Mesh] OR "HIV Seropositivity/diagnosis"[Mesh] OR "HIV Seropositivity/epidemiology"[Mesh] OR "HIV Seropositivity/mortality"[Mesh] OR "HIV Seropositivity/prevention and control"[Mesh] OR "HIV Seropositivity/statistics and numerical data"[Mesh] OR "HIV Seropositivity/transmission"[Mesh] OR "AIDS-Related Opportunistic Infections/diagnosis"[Mesh] OR "AIDS-Related Opportunistic Infections/epidemiology"[Mesh] OR "AIDS-Related Opportunistic Infections/mortality"[Mesh] OR "AIDS-Related Opportunistic Infections/prevention and control"[Mesh] OR "AIDS-Related Opportunistic Infections/statistics and numerical data"[Mesh] OR "AIDS-Related Opportunistic Infections/transmission"[Mesh] OR "Acquired Immunodeficiency Syndrome/diagnosis"[Mesh] OR "Acquired Immunodeficiency Syndrome/epidemiology"[Mesh] OR "Acquired Immunodeficiency Syndrome/mortality"[Mesh] OR "Acquired Immunodeficiency Syndrome/prevention and control"[Mesh] OR "Acquired Immunodeficiency Syndrome/statistics and numerical data"[Mesh] OR "Acquired Immunodeficiency; Syndrome/transmission"[Mesh]; #4 TB OR "Tuberculosis/diagnosis"[Mesh] OR "Tuberculosis/epidemiology"[Mesh] OR "Tuberculosis/mortality"[Mesh] OR "Tuberculosis/prevention and control"[Mesh] OR "Tuberculosis/statistics and numerical data"[Mesh] OR "Tuberculosis/transmission"[Mesh] OR "Tuberculosis, Pulmonary/diagnosis"[Mesh] OR "Tuberculosis, Pulmonary/epidemiology"[Mesh] OR "Tuberculosis, Pulmonary/mortality"[Mesh] OR "Tuberculosis, Pulmonary/prevention and control"[Mesh] OR "Tuberculosis, Pulmonary/statistics and numerical data"[Mesh] OR "Tuberculosis, Pulmonary/transmission"[Mesh] OR "Mycobacterium tuberculosis/diagnosis"[Mesh] OR "Mycobacterium tuberculosis/epidemiology"[Mesh] OR "Mycobacterium tuberculosis/statistics and numerical data"[Mesh]; #5"Coinfection/epidemiology"[Mesh] OR "Coinfection/mortality"[Mesh] OR "Coinfection/prevention and control"[Mesh] OR "Coinfection/statistics and numerical data"[Mesh] OR "Coinfection/transmission"[Mesh] OR HIV-TB OR HIV/TB OR TB-HIV OR TB/HIV. **#1 AND #2 AND ((#3AND#4)OR#5)** | 157 |
| **Web of Science**  **(All databases)** | Topic (TS). #1 Migrant OR immigrant OR foreign-born OR migration OR immigration OR emigrant OR emigration OR non-resident OR non-native OR cross-border OR “labour migration” OR “international migration”; #2 Albania OR Andorra OR Armenia OR Austria OR Azerbaijan OR Belarus OR Belgium OR "Bosnia Herzegovina" OR Bulgaria OR Croatia OR Cyprus OR "Czech Republic" OR Denmark OR Estonia OR Finland OR France OR Georgia OR Germany OR Greece OR Hungary OR Iceland OR Ireland OR Italy OR Kazakhstan OR Kosovo OR Latvia OR Liechtenstein OR Lithuania OR Luxembourg OR Macedonia OR Malta OR Moldova OR Monaco OR Montenegro OR Netherlands OR Norway OR Poland OR Portugal OR Romania OR Russia OR "San Marino" OR Serbia OR Slovakia OR Slovenia OR Spain OR Sweden OR Switzerland OR Turkey OR Ukraine OR "United Kingdom" OR Vatican; #3 HIV OR "Human Immunodeficiency Virus" OR AIDS OR "Acquired immune Deficiency Syndrome"; #4 TB OR tuberculosis OR "Mycobacterium tuberculosis"; #5 coinfection OR co-infection OR HIV-TB OR HIV/TB OR TB-HIV OR TB/HIV.**#1 AND #2 AND ((#3AND#4)OR#5)** | 438 |
| **Scopus** | Titles and Abstracts (ABS). #1 migrant OR immigrant OR foreign-born OR migration OR immigration OR emigrant OR emigration OR non-resident OR non-native OR cross-border OR "labor migration" OR "international migration"; #2 albania OR andorra OR armenia OR austria OR azerbaijan OR belarus OR belgium OR "Bosnia Herzegovina" OR bulgaria OR croatia OR cyprus OR "Czech Republic" OR denmark OR estonia OR finland OR france OR georgia OR germany OR greece OR hungary OR iceland OR ireland OR italy OR kazakhstan OR kosovo OR latvia OR liechtenstein OR lithuania OR luxembourg OR macedonia OR malta OR moldova OR monaco OR montenegro OR netherlands OR norway OR poland OR portugal OR romania OR russia OR "San Marino" OR serbia OR slovakia OR slovenia OR spain OR sweden OR switzerland OR turkey OR ukraine OR "United Kingdom" OR vatican; #3 coinfection OR co-infection OR hiv-tb OR hiv/tb OR tb-hiv OR tb/hiv; #4 hiv OR "Human Immunodeficiency Virus" OR aids OR "Acquired immune Deficiency Syndrome"; #5 tb OR tuberculosis OR "Mycobacterium tuberculosis". **#1 AND #2 AND (#3 OR (#4 AND #5)** | 151 |
